# Impaction of Mandibular First Molar by Tooth-Like Hard Tissue

**DOI:** 10.7759/cureus.59304

**Published:** 2024-04-29

**Authors:** Misa Ishiyama, Shunsuke Namaki, Hiroki Tamura, Kenchi Hase, Takashi Kikuiri

**Affiliations:** 1 Department of Pediatric Dentistry, Nihon University School of Dentistry, Tokyo, JPN; 2 Department of Oral and Maxillofacial Surgery, Nihon University School of Dentistry, Tokyo, JPN

**Keywords:** case report, spherical tooth-like hard tissue, permanent teeth impaction, first molar, eruption failure

## Abstract

Impaction of permanent teeth during the mixed dentition stage is relatively common in clinical practice, but impaction of mandibular first molars is rare. This case report presents an impaction of the mandibular first molar due to a tooth-like hard tissue lesion. An 8-year-old girl was diagnosed with an impacted mandibular first molar. The roots of the impacted molars were almost completely developed. A spherical tooth-like hard tissue with a diameter of approximately 2 mm was observed at the alveolar crest between the impacted mandibular first and second molars. The lesion causing the impaction was excised, and the first molar was fenestrated and allowed to erupt naturally. We showed that even if the tooth root is almost complete, natural eruption can be expected if the lesion is removed and space for eruption is secured.

## Introduction

Impaction of permanent teeth is a relatively common clinical occurrence [1]. The eruption of teeth is influenced by a complex interplay of various factors, including the growth of the tooth germ within the jawbone, the development of the tooth root, and the growth of the jawbone itself [2]. If these factors do not function properly, or if inhibitory factors are present, a tooth may become impacted. The incidence of impaction of permanent teeth ranges between 5.6% and 18.8% [3]. The most frequently impacted teeth are the third molars, followed by the upper canines, lower premolars, lower canines, upper premolars, upper central incisors, and lower second molars. The probability of a mandibular first molar becoming impacted is 0.01%, which is extremely rare [4]. It has been reported that once the tooth root is fully formed, the possibility of spontaneous eruption is significantly reduced [2]. In the present case, the roots of the mandibular first molar on the right were almost completely developed at the time of the visit. By removing the tooth-like hard tissue and fenestration, the impacted first molar was allowed to erupt naturally. This suggested that proper timing of diagnosis and treatment of impacted first molars are important for obtaining good results.

## Case presentation

**Figure 1:**
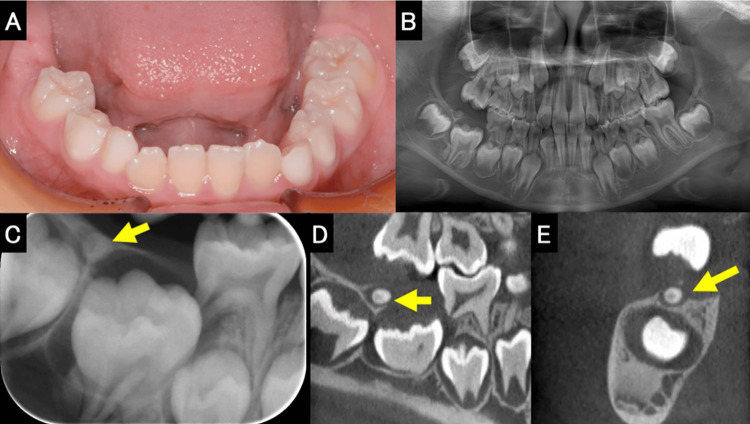
Intraoral photograph and radiographs (A) Intraoral photograph at first medical examination. The first molar on the right side of the mandible has not erupted. (B) Panoramic photograph. The roots of the first molars on both sides were complete. (C) Dental photograph at first medical examination. The yellow arrow indicates a spherical tooth-like hard tissue between the first and second molars on the right side of the mandible. (D, E) Dental CBCT images at first medical examination. The yellow arrow indicates a spherical tooth-like hard tissue. CBCT, cone beam computed tomography

**Figure 2:**
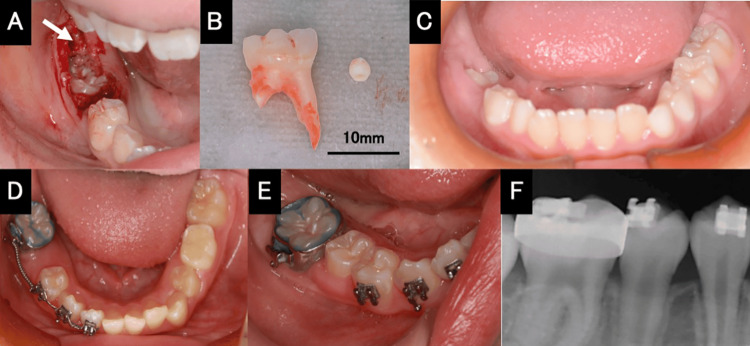
Treatment process (A) Intraoral photo immediately after surgical procedure. Extraction of a spherical tooth-like hard tissue and a second deciduous molar was performed, followed by the fenestration of the first molar. The white arrow indicates the extraction socket of the spherical tooth-like hard tissue. (B) Extracted second deciduous molar and tooth-like hard tissue. (C) Intraoral photograph eight months after surgery. The impacted first molar has almost erupted. The eruption space of the second premolar is limited. (D) To ensure space for the eruption of the second premolar, the first molar was moved distally using a compression force generated with an open-coil spring. (E) Eight months after initiating the distal move of the first molar, the eruption of the second premolar was completed. (F) Radiograph after eruption completion.

The patient, an 8-year-old girl, visited our department after her local doctor noted that her mandible first molar had not yet erupted. Intraoral examination showed that the molar had not erupted, while no crown-caused bulge could be palpated on the alveolar mucosa in the area corresponding to the first molar (Figure 1A). Dental and panoramic radiographic findings showed that the mandibular first molar on the right side was impacted, while the root was nearly complete (Figure 1B, 1C). A spherical opaque structure was observed at the alveolar crest edge between the first and second molars on the right side of the mandible. Approximately two-thirds of the distal root of the mandibular second deciduous molar had been resorbed (Figure 1C). Dental cone beam computed tomography (CBCT) revealed a spherical tooth-like hard tissue approximately 2 mm in diameter at the alveolar apex between the mandibular first and second molars (Figure 1D, 1E). The clinical diagnosis was an impaction of the first molar on the right side of the mandible and an impaction of tooth-like hard tissue between the first and second molars on the right side of the mandible. After infiltration anesthesia, the alveolar bone corresponding to the first molar on the right side of the mandible was removed, and the dental follicle-like soft tissue and tooth-like hard tissue were extracted. Thereafter, the alveolar bone around the mandibular right first molar was removed and a fenestration was performed, followed by the extraction of the mandibular right second deciduous molar (Figure 2A). The extracted hard tissue was approximately 2 mm in diameter (Figure 2B). Intraoral examination one month after surgery revealed a portion of the crown of the first molar on the right side of the mandible. Three months after surgery, the crown of the mandibular right first molar was observed to be erupting in the normal direction (Figure 2C). Nine months after surgery, the mandibular right first molar had almost completely erupted and was confirmed to be occluding with the opposing tooth (Figure 2D). Thereafter, because of a lack of space for the eruption of the mandibular right second premolar, the mandibular right first molar was moved distally. Accordingly, space was created for the eruption of the mandibular right second premolar, and the eruption was completed (Figure 2E, 2F). The procedure was explained to the parents of the patient, who signed an informed consent allowing treatment procedures and publication of data.

## Discussion

First molars play a crucial role in establishing normal occlusion. Therefore, when a first molar becomes impacted, it can cause not only malocclusion but also affect the development of the jawbone, leading to distortion of the entire dentition and potentially widespread malalignment [5]. Accordingly, the correct eruption of impacted first molars at the right time and in the right position is very important. When determining the timing of treatment, several factors need to be considered, including the age of the patient, the presence or absence of a lesion, the position of impaction within the jawbone, the condition of the opposing teeth, and the degree of root development of the impacted first molar [6]. In addition, if the second molars erupt while the first molars remain impacted, the second molars may shift mesially, closing the eruption space for the first molars. Ideally, treatment should be performed before the eruption of the second molars. Treatment methods for impacted teeth are broadly divided into follow-up observation, removal of the cause, induction of eruption, and tooth extraction [7]. First, the possibility of inducing eruption into the dentition should be considered. When considering guidance into the dentition, removal of an existing lesion should be performed; if no lesion exists, eruption should be encouraged. After fenestration, two options are available: to observe the progress in hopes of spontaneous eruption, or to actively induce eruption through traction treatment [8]. There is no consensus on the decision and timing of performing traction treatment; however, if the tooth root is incomplete, eruption output can be expected. Hence, many studies have reported tooth eruption with fenestration alone, with the tooth axis being significantly tilted, causing eruption. However, various other studies have reported that in cases of an abnormality in the direction of the tooth or completed tooth root formation, traction treatment was immediately performed to actively induce eruption [9].

## Conclusions

In this case, in which the root of the tooth was almost complete, by removing the spherical tooth-like hard tissue lesion and performing fenestration, the tooth erupted naturally without the requirement of traction treatment. In cases where the root of the tooth is almost complete, natural eruption can be expected if the lesion is removed and space for eruption is secured.
